# Diagnostic paths and service needs of children with autism spectrum disorder and with other neurodevelopmental disorders in Bulgaria

**DOI:** 10.3389/fpsyt.2022.937516

**Published:** 2022-12-22

**Authors:** Mihaela D. Barokova, Ana Andreeva-Sapundzhieva, Elena Andonova, Galina Markova-Derelieva, Arun Karpur

**Affiliations:** ^1^Department of Cognitive Science and Psychology, New Bulgarian University, Sofia, Bulgaria; ^2^Center for Social Rehabilitation and Integration, Sofia, Bulgaria; ^3^Department of Health Care and Social Work, New Bulgarian University, Sofia, Bulgaria; ^4^Autism Speaks, Princeton, NJ, United States

**Keywords:** diagnosis, service needs, Bulgaria, ASD, neurodevelopmental disorders

## Abstract

**Background:**

Currently, there are no official statistics about the number of children with developmental disorders in Bulgaria. This is the first systematic investigation of the needs, access to services, and priorities of families of children with developmental disorders in the country.

**Aims:**

The study aims to: (1) characterize the needs of children with developmental disorders in Bulgaria; (2) to compare the needs and access to services of children with Autism Spectrum Disorders (ASD) and other neurodevelopmental disorders (oNDD); (3) and to examine the daily burden of their caregivers and how it varies based on their demographic characteristics, such as income and education.

**Methods:**

We used an online family needs assessment survey to collect data from caregivers of children with developmental disorders in Bulgaria between April and July 2020. 195 parents of children with ASD and 73 parents of children with oNDD completed the questionnaire.

**Results:**

Children with ASD waited longer than children with oNDD to receive a diagnosis. Caregivers in the ASD group also expressed first concerns about their child’s development when their children were older and for different reasons than caregivers in the oNDD group. There were no significant differences between groups in service encounters, including access to and delay of medical, counseling, and educational services, with approximately 50% of all caregivers experiencing some delay and/or difficulties in access to services. There were no associations between access to services and caregiver education and family income, with the exception of higher education being linked to receiving a diagnosis earlier for the oNDD group.

**Discussion:**

This study has three main findings: (1) children with ASD and children with oNDD in Bulgaria have different needs and paths to diagnosis; (2) nevertheless, children in both groups experience similar challenges in accessing medical, counseling, and educational services, regardless of their demographic characteristics; and (3) parents’ priorities focus on education, counseling, and medical support, protecting children’s basic rights, and raising awareness. A comparison of our findings to past research in the region shows a relative improvement in diagnostic services with families not having to travel outside their city to receive a diagnosis. Based on our findings, we provide specific recommendations for changes in services and policy.

## 1 Introduction

Over the last decade, a concerted effort has been made to shed light on the needs of families of children with developmental delays, disorders, and disabilities in low- and middle-income countries in Europe. Specifically, the establishment of the Southeast European Autism Network (SEAN) in 2010 as part of the Autism Speaks Global Autism Public Health Initiative helped raise awareness about autism spectrum disorders (ASD) and other neurodevelopmental disorders (oNDD) (1). The increasing number of scientific meetings, conferences, clinical and parent workshops combined with the rapidly increasing number of parent-led organizations and non-governmental organizations suggests that there is a growing awareness and demand for services for children with disabilities in the region. Nevertheless, still very little is known about the specific needs and challenges families of children with developmental disorders^1^ experience. To address this gap the present study reports primary data on the needs and daily burdens of families of children with developmental delays, disorders, and disabilities in Bulgaria collected online between April and July 2020.

### 1.1 Health care in Bulgaria

In order to situate our study in the context of services for children with developmental disorders provided in Bulgaria, we provide a brief overview of the country’s health care system. In Bulgaria, there is a National Health Insurance Fund, whose main goal is “to ensure and guarantee free and equal access to health care for insured persons – through a set of types, scope and volume healthcare activities, as well as free choice of an executor who has a contract with a regional health insurance fund” (2). The fund is financed through mandatory taxes, specifically for health insurance. People can also enroll in private health insurance plans for additional benefits. In the context of seeking a diagnosis for a neurodevelopmental disorder, with referrals from a pediatrician or a general practitioner, families can receive consultation from a child psychiatrist and get medical tests done for free or for a subsidized fee. Once the child receives a formal diagnosis and a referral, they can access state-funded service providers for treatment and intervention. Families can also seek additional services through paid providers with varying fees. The wait times for accessing these services vary, and so do the kinds of services that are available at a specific resource center (counseling, speech therapy, occupational therapy, physical therapy, etc.).

### 1.2 The needs and experiences of children with developmental disorders in Bulgaria

In Bulgaria, there are no publicly available data on the total number of children with disabilities and their breakdown by diagnosis and specific medical, counseling, and educational needs. In the fall of 2019, the Bulgarian UNICEF office published statistics – provided by the Ministry of Education and Science – showing that during the 2019−2020 school year, the number of children with disabilities and with specific educational needs was over 25,000 (2.5% of all school-age children in Bulgaria in 2019), of whom 15,000 presented with difficulties in communication (3). At that time, additional 10,000 children with disabilities (1% of all school-age children in Bulgaria in 2019) were reported to not be enrolled in an educational institution. Based on these statistics, there are close to 35,000 school-aged children with disabilities in Bulgaria, which makes up 3.5% of all school-age children in the country (*N* = 994 667) based on data from the National Statistical Institute. This percentage would potentially be higher when infants and toddlers with developmental disorders are included. Nevertheless, there is no published systematic investigation of the needs and priorities of these children and their families.

Of the few published, peer-reviewed papers and books on the topic, to our knowledge, only one used primary data from families (4), while the rest provide useful, albeit broad overview of the available services (5), relevant policy and governmental structures/mechanisms and the history and development of intervention, therapeutic, and research practices in the country (e.g., 6, 7). Daniels et al. (4) reported results from a parent questionnaire administered to caregivers of children with ASD (*N* = 147) in Bulgaria between 2013 and 2015. Based on their responses, parents expressed a first concern about their children when they were on average 24.7 months old, and the average age of diagnosis was 46.6 months. In addition, half of the parents reported traveling over 100 km to obtain their child’s diagnosis. Parents reported on utilizing services, with 94% receiving speech and language therapy in the past and 83% receiving it at the time of assessment. In terms of receiving other supports for their children, 70% reported receiving government assistance, and 30% reported relying on advocacy groups and 42% of parents reported receiving training or assistance in general. Many parents (43%) endorsed feelings of helplessness in providing care for their children (4).

In terms of paths to a formal diagnosis, families’ experiences vary. Based on anecdotal reports from parents, some seek out a diagnosis themselves by signing their children up for a diagnostic assessment. Other parents report that it is their pediatrician that encouraged them and provided them with a referral for a child psychiatrist. In Bulgaria, only a child psychiatrist can provide a formal diagnosis of ASD or other neurodevelopmental disorders. Typically, the diagnostic evaluation includes an assessment by a child psychiatrist, a neurologist, and a clinical psychologist among other professionals. An autism diagnosis is typically not given before the age of 3 years. Yet, other parents prefer to undergo an informal diagnostic evaluation that does not lead to the issuance of an official document. Overall, diagnostic experiences vary, but to date, there is no published formal evaluation of age of diagnosis, diagnostic procedures and protocols across clinics, and duration of the diagnostic process.

When it comes to access to services, there is also a lot of variation across families and providers. Access is greatly influenced by how their children’s disorders are conceptualized. In the past, the medical model of disability identification was central (8). As a result, children were classified based on their disability and they were more likely to be placed in specialized institutions. In recent years, there has been a transition toward a more social model of conceptualizing these children’s conditions and consequently educational experiences by focusing on their potential for learning and change.

In addition to the way developmental disorders are conceptualized, community awareness and attitudes could also influence the experiences of children with developmental disorders. An independent report on parental attitudes published by the Center for Inclusive Education demonstrated that parents’ subjective level of acceptance of children with developmental disorders in the classroom varied by their diagnosis (9). Children with motor difficulties and speech-language disorders were accepted by 66% and 63% of respondents, respectively. Parents were much less likely to accept the integration of children with ASD in the classroom (33%) and even less likely to accept children with intellectual disabilities and multiple comorbid conditions (13−16%). The authors attributed these differences to lack of awareness about these children’s conditions, their educational needs, and how they can be addressed in the classroom.

Although informative, these studies were all conducted over 5 years ago. Since then, many new government initiatives have taken place to address the needs of these children with developmental disorders and their families.

### 1.3 Major initiatives and policy changes in the last 5 years

As of the beginning of 2019, the Persons with Disabilities Act guarantees institutional/governmental support for persons with disabilities through means of medical, professional, social, occupational, and psychological rehabilitation, education and professional training, and access to information among others (10). Furthermore, this act regulates a monthly financial assistance for individuals with disabilities and imposes new obligations on employers to promote their employment.

Specifically targeting children’s welfare and rights, the National Strategy for Children (2019−2030) was drafted, albeit delayed in its formal approval and implementation (11). This national strategy follows the aims and priorities set forth by the UN Convention on Children’s Rights focusing on ensuring the children’s rights to life and development. In the meantime, the Social Services Act that went into effect on 1 July 2020 guarantees individuals’ right to social services, including therapy and rehabilitation, assistant services, and day and residential care (12). It also emphasizes the need for providing services not only for the child but for its family as a unit as well, which had not been emphasized in the past.

Perhaps, most progress has been made in the educational sector, specifically as it pertains to inclusive education. As of 2017, the ordinance on inclusive education regulates mandatory screening of all children between 3 and 3.5 years of age upon enrollment in kindergarten (13). Based on the screening and evaluation, an individual educational plan is to be designed. This new regulation complements earlier reforms of the Public Education Act (Article 21) now part of the Pre-School and School Education Act, which mandated the inclusion of children with specific educational needs in the schools, and allowed for the development of individual educational plans, while the child still attended school with typically developing peers (14).

Despite reforms in laws, regulations, and national strategies, whether and how they are implemented in practice and what their effects are on the daily lives of families with children with disabilities has not been examined systematically. The only document providing an evaluation of how the country ensures children’s welfare is the yearly publication of the National Network for Children’s Report Card (15). The Network is an alliance of organizations united by the common goal to promote and protect the rights of children in Bulgaria. In their yearly report card, they summarize and present the evaluations of various organizations, clinicians, teachers, and even children on whether and how the governmental structures have implemented laws and policies. The report focuses on key aspects of children’s welfare including early child development, health, education, and protection of children’s rights among others. Their evaluation claims that little progress has been made in early child development with key issues being the lack of enough trained professionals and lack of coordination between providers and governmental agencies. The report suggests that most progress has been made in the educational sector due to strong political commitment. Despite the well-developed plans and strategies for inclusive education, there is still a dearth of resource teachers (trained educators at school that work one-on-one with children inside and outside of the classroom) to address the needs of children with special educational needs in the schools (15).

### 1.4 Early intervention in Bulgaria

Although the policy has changed over the past 5 years, special note should be taken on the state of early intervention in the country. Based on data collected between 2018 and 2019, UNICEF published a report on the topic (16). Data were collected through focus groups and online questionnaires with parents, teachers, psychologists, speech-language pathologists (SLPs), kindergarten staff, and other professionals involved in early intervention. Based on the Ordinance on Prophylactic Examinations and Dispanserization Services from November 2016 (17), every general practitioner and pediatrician in Bulgaria is required to monitor the development of children visiting their practice. Based on the UNICEF report, indeed over 70% of parents report that their pediatrician monitored the physical development of their child (in terms of weight and height), but only 12% reported that their child’s learning and behavior had been evaluated (16). When medical professionals themselves were asked about barriers to developmental screening, 51.4% of them indicated that they do not know what instruments to use and that instruments were lacking, and 62.9% reported lack of trained personnel to do the screening. In addition, when early intervention service providers were asked about screening their clients, only 28% reported conducting screening at all, and only 15% listed a specific instrument that they used. Among the used listed instruments were the Ages and Stages Questionnaire (ASQ; 18), Denver Developmental Screening Test-II (19), Modified Checklist for Autism in Toddlers, Revised (M-CHAT-R; 20), and Vasilka Manova-Tomova (21). In addition, according to the report, 38% of professionals have no experience working with children under the age of 3 years (16). In addition, 44% of surveyed kindergarten and nursery staff reported having no experience working with children with developmental disorders or disabilities. Overall, early intervention is extremely limited in the country and many screening and assessment instruments have yet to be normed and validated.

### 1.5 Current study

Regardless of these reports and evaluations, there is still a need for a systematic investigation of the needs and priorities of families of children with developmental disorders, delays, and disabilities, and how the system of services interacts with their demographic characteristics and diagnosis. The present study addressed this gap in past research by collecting an online caregiver needs survey from parents of children with developmental disorders in Bulgaria. The study had three aims: (1) to characterize the needs of children with developmental disorders in Bulgaria; (2) to compare the needs and access to services of children with ASD and other neurodevelopmental disorders; (3) to examine the daily burden of their caregivers and how it varies based on their demographic characteristics, such as income and education.

## 2 Materials and methods

### 2.1 Survey

The survey used in the present study was adapted from the Caregiver Needs Survey developed by Autism Speaks (22). The aim of the original survey was to assess the needs of families, who have a child with a confirmed ASD diagnosis. For the present purposes, the survey was adapted for use with families of children with a confirmed diagnosis of developmental delays, disorders, and/or disabilities more broadly. We acknowledge that by including only children who have a confirmed diagnosis we are not able to assess the needs of families who are unable or unwilling to seek diagnostic services. Assessing the needs of such families would require a very different methodological and recruitment approach. Nevertheless, this study is a first step toward understanding the family needs of children with neurodevelopmental disorders in Bulgaria. The survey consisted of three parts.

In ***Part 1*,** we collected demographic information about the respondent, including age, sex, relationship to the child, education, employment status, marital status, and household income. Similar information was also collected about the respondent’s partner, if applicable.

In ***Part 2*,** we collected information about the child’s characteristics and the caregiver’s first concerns. Questions covered the reasons why the caregiver sought a diagnosis, the age of diagnosis, and the current diagnosis of the child. We also asked caregivers about what they thought caused their child’s condition (e.g., genetic predisposition, vaccines, act of God, traumatic experiences in early childhood, unknown, etc.).

In ***Part 3*,** we collected information about service encounters and the caregiver’s needs and perceptions. Questions covered whether in the past year the child received medical and counseling services and if not, why. We collected information about whether the child attended kindergarten or school, what type, and whether the school knew about their diagnosis. Caregivers were asked about the availability of parent trainings and workshops in their region and about whether they experienced difficulty in finding information about their child’s condition. We also asked caregivers to pick the 3 main challenges associated with their child’s condition, the three main challenges associated with their access to care, and the three main priorities when receiving support.

At the end of the survey, we added a comment box, not included in the original Caregiver Needs Survey, in order to give respondents the opportunity to share their experiences that might not have been reflected in the survey questions. The prompt for it was as follows: “Comments, recommendations, questions (e.g., What kind of professionals do you need access to? What should the main priorities be when changing current policy related to ensuring services for children with developmental disorders and their families?).”

### 2.2 Preparation of the Bulgarian version

The survey was first translated into Bulgarian by a licensed translator. The translation was then reviewed and adapted to the Bulgarian context by two clinicians, who work with children with developmental disorders and their families, and two researchers with experience in developing instruments. This adapted version was then presented to two separate focus groups. The focus groups were facilitated by a clinician and a researcher and consisted of five primary caregivers of children with developmental disorders (primarily ASD) of varying age. The focus groups were presented with the survey questions verbally and were asked to provide feedback. Based on the received feedback, the wording of some questions and their associated responses was changed to better reflect the experiences of caregivers. In particular, more options were included in the questions about caregiver’s employment status to reflect individual experiences of being the primary caregiver, as well as working full-time. Yearly income was changed to monthly income. For the caregivers’ perceptions of the causes of their child’s diagnosis, we added a medical/doctor’s error/malpractice option. With regard to who encouraged the caregiver to seek a diagnosis, we added the option of “encouragement/recommendation from pre-K and kindergarten personnel” again based on the focus group feedback.

### 2.3 Procedure

In Bulgaria, there is no population-based sampling of children with developmental disorders, so a sample of convenience was used. Caregivers of children with developmental delays, disorders, and disabilities were recruited through listservs of service providers in the big cities around the country, through posts on NGO websites and parent groups on social media platforms, and through posts on news and media outlets (radio, newspapers, etc.). The survey was sent out in the form of a link.

Respondents had to read and fill out an online consent form prior to starting their participation. The survey took between 30 and 40 mins to complete. Data collection took place between April and July 2020.

The Research Ethics Committee of the Cognitive Science and Psychology Department at the New Bulgarian University approved this project prior to respondent participation.

### 2.4 Participants

While the survey was active online, 422 respondents opened it, 7 did not give consent to participate, and 98 gave consent but did not fill out any questions, which left us with a sample of 317 respondents. Out of them, 7 did not indicate their relationship to the child and 5 indicated that they were a clinician, resource teacher, and/or a medical professional working with children with developmental disorders. These 12 respondents were excluded, which left us with a final sample of 305 respondents.

### 2.5 Data analysis plan

The data from the survey was collected using SurveyMonkey (SurveyMonkey Inc., San Mateo, CA, United States). Data were then imported and analyzed using the Statistical Package for Social Sciences Version 26.0 (SPSS 26.0). Data analysis included descriptive statistics such as frequency distributions, and inferential statistics comparing responses across child diagnostic status and respondent’s income and education as proxy for SES.

## 3 Results

### 3.1 Child diagnosis and groups

To categorize our sample by diagnosis, we asked respondents to report their child’s most recent diagnosis, with the option of including more than one. Table 1 includes the distribution of diagnoses across children. Note that clinical diagnoses in Bulgaria are based on the International Statistical Classification of Diseases and Related Health Problems (ICD-10; 23). The most common diagnosis in our sample was autism (27.9%), followed by PDD (24.9%) and ASD (16.7%) as defined by the ICD-10. Under other/written in diagnoses, responses included hydrocephaly (*N* = 4), Asperger’s syndrome (*N* = 1), childhood autism (*N* = 1), Coffin-Siris Syndrome (*N* = 1), Prader-Willi Syndrome (*N* = 1), Down Syndrome (*N* = 1), Rett Syndrome (*N* = 1), and dyslexia (*N* = 1) among others. We also examined the overlap across diagnostic categories. 63.9% of children in our sample had an autism diagnosis, including autism, autism spectrum disorder, pervasive developmental disorder, pervasive developmental disorder – not otherwise specified, Asperger’s Syndrome, and/or childhood autism. The second most common category of diagnoses was a mix with 13.4% of children receiving multiple diagnoses across autism, ADHD, intellectual disability, cerebral palsy, and epilepsy. In terms of the distribution of total number of diagnoses in our sample, the majority of children (61%; *N* = 186) had a single diagnosis, 18.7% (*N* = 57) had two, and 8.1% (*N* = 25) had 3 or more.

**TABLE 1:** Distribution of diagnoses and total number of diagnoses in the full participant sample (*N* = 305).

Characteristic	*N*	% Out of total
**Diagnosis (could choose more than one)**		
Autism	85	27.9
Pervasive Developmental Disorder (PDD)	76	24.9
Autism Spectrum Disorder (ASD/PAC)	51	16.7
Pervasive Developmental Disorder – Not Otherwise Specified (PDD-NOS)	35	11.5
Developmental delay	29	9.5
Intellectual disability	28	9.2
ADHD	23	7.5
Written in diagnosis	23	7.5
Cerebral palsy	22	7.2
Epilepsy	15	4.9
No diagnosis	11	3.6
Did not respond	27	8.9
**Distribution of diagnoses**		
Autism, ASD, PDD, PDD-NOS, DD, Asperger, Childhood Autism	195	63.9
Developmental delay	5	1.6
ADHD	8	2.6
Intellectual disability	2	0.7
Cerebral palsy	11	3.6
Epilepsy	1	0.3
Across categories – combined	41	13.4
Other	10	3.3
No diagnosis	10	3.3
Did not respond	27	8.9
**Number of diagnoses**		
0	37	12.1
1	186	61.0
2	57	18.7
3	15	4.9
4	8	2.6
5	1	0.3
6	1	0.3

For the purposes of the following analyses, we divided participant responses into two groups based on child diagnosis. The first group, hereby called ASD Group, comprises respondents (*N* = 182), whose child had an autism and/or related diagnosis including childhood autism, autism spectrum disorder, pervasive developmental disorder, pervasive developmental disorder – not otherwise specified, and/or Asperger’s Syndrome. The second group, hereby called oNDD Group, comprises of respondents (*N* = 74), whose child had any of the other neurodevelopmental disorders including ADHD, intellectual disability, cerebral palsy, epilepsy, different syndromes, or multiple diagnoses spanning across these categories. Children who received both an ASD diagnosis and a diagnosis that belonged to the oNDD category were classified as oNDD. This was done because we hypothesized that a child who had an ASD and an additional NDD diagnosis, for example, epilepsy or cerebral palsy, would have different needs and would try to access different services as compared to a child who only had an ASD diagnosis.

### 3.2 Needs of the child

First, we focused on characteristics that could directly affect the child’s needs and access to services including family demographic information and child characteristics.

#### 3.2.1 ASD group

##### 3.2.1.1 Family characteristics

The majority of respondents were female (94%) between the ages of 36 and 45 (56%) and identified themselves as the biological mother (89%) and primary caregiver (89.6%) of the child for whom they were filling out the survey (see Table 2). In terms of race and ethnicity, which was operationalized as native language, the vast majority of respondents (98.9%) identified as Bulgarian.

**TABLE 2:** Caregiver demographic characteristics across the participant groups.

	ASD group *N* = 182		oNDD group *N* = 74	
Characteristics	*N*	Valid%	*N*	Valid%
**Gender**				
Female	171	94.0	74	100
Male	11	6.0	−	−
**Age (in years)**				
18−25	3	1.6	−	−
26−35	33	18.1	17	23.0
36−45	102	56.0	38	51.4
46−55	37	20.3	18	24.3
56−65	1	0.5	−	−
>65	6	3.3	1	1.4
**Relationship to child**				
Biological mother	162	89.0	68	91.9
Biological father	9	4.9	−	−
Adoptive mother	3	1.6	3	4.1
Foster parent	0	0	2	2.7
Grandparent	5	2.7	1	1.4
Other family member	3	1.6	−	−
**Primary caregiver of the child**				
Yes	163	89.6	69	93.2
No	19	10.4	5	6.8
**Native language**				
Bulgarian	180	98.9	73	98.6
Other	2	1.1	1	1.4
**Family status**				
Married or living together	152	83.5	65	87.8
Widowed, divorced or separated	17	9.3	3	4.1
Single	13	7.1	5	6.8
Did not respond	−	−	1	1.4
**Education *Simplified***				
School education	38	20.9	9	12.2
Undergraduate (classes and/or degree)	57	31.3	33	44.6
Graduate degree	87	47.8	32	43.2
**Income *Simplified***				
<1220l v	71	39.0	27	36.5
1220–1830 lv	48	26.4	20	27.0
>1830 lv	52	28.6	23	31.1
Did not respond	11	6.0	4	5.4
**Employment *Simplified***				
Full-time	72	39.6	27	36.5
Part-time	31	17.0	13	17.6
Other	77	42.3	34	45.9
Did not respond	2	1.1	−	−

When characterizing the families’ socio-economic status (SES), we looked at the education of the respondent and the family monthly income (in leva). The majority of respondents had an undergraduate degree or higher (79.1%). Even though the caregiver education was skewed toward higher attained degrees, the household monthly income resembled a normal distribution with 39% of respondent earning 1.220 lv, which is at or below country average (1.148 lv; 24), 26.4% earning between 1.220 and 1830 lv per month, and 28.6% earning over 1.830 lv per month. In terms of employment, 39.6% reported working full-time, and 17% reported working part-time.

##### 3.2.1.2 Child characteristics

We did not collect age and gender information on the children. However, we have information about the children’s kindergarten or school enrollment. Based on it, 30.8% (*N* = 56) children were under the age of 6 and 58.2% (*N* = 106) of the children were school-age or older than 6 years (see Table 3). Next, we examined caregivers’ reasons for first concern regarding their child’s development. 75.3% of caregivers chose communication difficulties, 66.5% chose social challenges, and 58.2% chose restricted and repetitive behaviors (see Table 3). Respondents were the ones to first notice and express concern about their child’s development in 67% of cases, followed by other family members (12.6%), and spouses (4.4%). The majority of children (58.2%) were between 12 and 24 months at the time when the first concern about their development was noticed/expressed. 13.2% of children were under the age of one and 28.6 were between 2 and 6 years of age when someone expressed a concern about them.

**TABLE 3:** Caregiver-reported child characteristics, reasons for first concern, and diagnosis information.

Question	ASD group (*N* = 182)		oNDD group (*N* = 73)	
	***N***	**%**	***N***	**%**
**Type of School/Kindergarten**				
Kindergarten	56	30.8	18	24.3
State School	56	30.8	22	29.7
Private School	4	2.2	5	6.8
Specialized School	22	12.1	13	17.6
Not enrolled	11	6.0	8	10.8
Other	9	4.9	3	4.1
Did not respond	24	13.2	5	6.8
**Proxy for age**				
Under 6	56	30.8	18	24.3
Over 6	106	58.2	45	60.8
Unknown	20	11.0	11	14.9
**Reasons for first concern**				
Communication difficulties	137	75.3	39	52.7
Social challenges	121	66.5	18	24.3
Repetitive behaviors	106	58.2	13	17.6
Motor difficulties	35	19.2	42	56.8
Medical difficulties	36	19.8	23	31.1
Behavioral difficulties	51	28.0	24	32.4
**First to notice the concern**				
I was	122	67.0	43	58.1
My spouse/domestic partner	8	4.4	3	4.1
Other family member	23	12.6	6	8.1
Health care provider	11	6.0	16	21.6
Teacher	9	4.9	4	5.4
Other	9	4.9	2	2.7
**Child’s age when first concern was noticed**				
0–3 months	4	2.2	22	29.7
3–6 months	4	2.2	9	12.2
6–12 months	16	8.8	13	17.6
12–18 months	63	34.6	11	14.9
18–24 months	43	23.6	6	8.1
24 months–3 years	40	22.0	9	12.2
3–6 years	12	6.6	3	4.1
7–12 years	−	−	1	1.4
**Child’s age at diagnosis**				
<1 year old	−	−	34	45.9
1–3 years	106	58.2	25	33.8
4–8 years	69	37.9	14	18.9
9–12 years	4	2.2	1	1.4
13–17 years	−	−	−	−
>18 years	1	0.5	−	−
No diagnosis	2	1.1	−	−
**Primary factor to pursue diagnosis**				
Symptoms worsened	75	41.2	33	44.6
Encouragement from family members and friends	56	30.8	15	20.3
Encouragement from kindergarten teacher	26	14.3	10	13.5
Encouragement from community leaders to seek medical evaluation	6	3.3	3	4.1
Public service announcement that encouraged pursuit of medical advice	2	1.1	−	−
Previously unavailable healthcare services became available	4	2.2	2	2.7
Other (written in)	33	18.1	21	28.4
**Number of diagnoses**				
1	150	82.4	32	43.2
2	30	16.5	23	31.1
3	2	1.1	10	13.5
4	−	−	7	9.5
5	−	−	1	1.4
6	−	−	1	1.4
				

With regard to children’s diagnosis, 58.2% received a diagnosis by the age of 3 years, and additional 37.9% received a diagnosis by the age of 8 years, with less than 5% receiving a diagnosis past that age. The most common factors that lead to the caregiver seeking a diagnosis for their child were that the child’s symptoms worsened (41.2%) and that family members or friends encouraged it (30.8%), followed by encouragement from a kindergarten teacher (14.3%). Interestingly, 18.1% of parents selected “other reasons” for pursuing a diagnosis for their child, and the majority of them wrote in their response explaining that it was their desire to help their child develop and achieve their full potential that encouraged them.

There were no significant associations between caregiver education or income with age of first concern or with age of diagnosis.

#### 3.2.2 oNDD group

##### 3.2.2.1 Family characteristics

All family demographic characteristics for the oNDD group can be found in Table 2.

##### 3.2.2.2 Child characteristics

All child characteristics can be found in Table 3.

When examining how family income and caregiver education were related to the diagnostic experiences, no significant associations between caregiver income with age of first concern or age of diagnosis were found. There was a statistically significant association between caregiver education and child age of diagnosis, however. Parents with a higher attained degree were more likely to have a child who was diagnosed earlier [χ^2^(6, *N* = 74) = 19.39, *p* = 0.004].

#### 3.2.3 ASD vs. oNDD comparison

When comparing the ASD and oNDD Groups, there were no differences between groups in terms of demographic characteristics such as parent education, monthly income, or employment status. There were some group differences in child characteristics, however. Caregivers in the ASD Group were more likely to choose communication difficulties [χ^2^(1, *N* = 256) = 12.48, *p* < 0.001], social challenges [χ^2^(1, *N* = 256) = 37.68, *p* < 0.001], and repetitive behaviors [χ^2^(1, *N* = 256) = 34.99, *p* < 0.001] as reasons for first concern, while caregivers in the oNDD group were more likely to pick motor difficulties [χ^2^(1, *N* = 256) = 35.23, *p* < 0.001]. Furthermore, there was a marginally significant difference in who expressed first concern about the child across the diagnostic group with caregivers in the oNDD Group being more likely to select a medical professional than parents in the ASD group [χ^2^(5, *N* = 256) = 14.36, *p* = 0.013].

There was a statistically significant association between diagnostic group and age of first concern as well [χ^2^(7, *N* = 256) = 72.53, *p* < 0.001]. Caregivers in the oNDD Group were more likely to notice a first concern about their child’s development when the child was younger compared to caregivers in the ASD group. A similar pattern was observed with regard to child’s age at diagnosis [χ^2^(5, *N* = 256) = 97.04, *p* < 0.001]. There were no group differences in reasons to pursue a diagnosis.

### 3.3 Daily burden of caregiver

Next, we focused on factors that contribute to the daily burden of caregivers, including caregiver knowledge and accessibility to treatments and services.

#### 3.3.1 ASD group

When asked about their beliefs about the causes of their child’s condition, 36.8% of caregivers reported that there were no known causes, 33.5% selected vaccinations as the cause, and 9.9% selected genetics or hereditary causes, while the remaining responses spanned across act of God, traumatic experiences early in life, bad luck, and medical error (see Table 4). There was a statistically significant association between caregivers’ beliefs about the causes of their child’s condition and their education [χ^2^(16, *N* = 182) = 31.18, *p* = 0.013]. Based on visual inspection of the distribution of responses across the three education categories, it appears that the caregivers, who had attained higher education degrees were more likely to select “unknown causes” and “genetic causes,” while the distribution across the “vaccines” response was similar across education sub-groups. There was no association between beliefs and family income.

**TABLE 4:** Caregiver-reported beliefs, paths to diagnosis, and access to services.

	ASD group (*N* = 182)		oNDD group (*N* = 74)	
**Question**	***N***	**%**	***N***	**%**
**Beliefs about the causes of their child’s condition**				
Act of God or supreme being	1	0.5	2	2.7
Traumatic experiences early in life or in womb	16	8.8	15	20.3
Genetics or hereditary in some families	18	9.9	13	17.6
Cold, rejecting parents	−	−	3	4.1
Vaccinations	61	33.5	10	13.5
Bad luck or a curse from one’s past life	3	1.6	1	1.4
Medical error/malpractice	7	3.8	8	10.8
No known causes	67	36.8	14	18.9
Other	8	4.4	7	9.5
Did not respond	1	0.5	1	1.4
**Number of children with dev. disorder**				
None	5	2.7	2	2.7
One	163	89.6	69	93.2
Two	11	6.0	3	4.1
Three or more	1	0.5	−	−
Did not respond	2	1.1	−	−
**Number of adults with disorder**				
Yes	19	10.4	9	12.2
No	163	89.6	65	87.8
**How far did you have to travel to obtain a diagnosis?**				
Within my town/village	121	66.5	48	64.9
A few towns/villages away	26	14.3	11	14.9
Had to travel into another province	31	17.0	10	13.5
I traveled outside the country	3	1.6	5	6.8
Did not respond	1	0.5	−	−
**How long did you have to wait between your initial pursuit of a diagnosis and the ultimate confirmation of a clinical diagnosis for your child?**				
Less than a month	40	22.0	30	40.5
1–3 months	53	29.1	28	37.8
3–6 months	45	24.7	5	6.8
6–12 months	17	9.3	1	1.4
Over a year	25	13.7	7	9.5
We still have not received a diagnosis	2	1.1	3	4.1
During the past year, did your family have any difficulties or delays in getting medical services for your child for any of the following reasons?*				
There were no difficulties or delays	85	46.7	37	50.0
Delayed because of ineligibility	13	7.1	7	9.6
Delayed because of lack of information about services	31	17.0	10	13.5
Delayed because of lack of services in our area	15	8.2	8	10.8
Delayed because of long wait times	39	21.4	13	17.6
Delayed because of unreasonable costs	19	10.4	7	9.5
No services available	16	8.8	9	12.2
Other	9	0.5	5	6.7
**Has your child ever received counseling services to meet his/her behavioral needs?**				
Yes	153	84.1	65	87.8
No	4	2.2	2	2.7
I do not know	3	1.6	1	1.4
Did not respond	22	12.1	6	8.1
During the past year, did your family have any difficulties or delays in getting counseling services for your child for any of the following reasons?*				
There were no difficulties or delays	103	56.6	39	52.7
Delayed because of ineligibility	7	3.8	6	8.1
Delayed because of lack of information about services	12	6.6	7	9.5
Delayed because of lack of services in our area	10	5.5	7	9.5
Delayed because of long wait times	16	8.8	3	4.1
Delayed because of unreasonable costs	18	9.9	9	12.2
No services available	9	4.9	7	9.5
Other	9	4.9	5	6.8
**Does your child’s school offer any additional academic support for children with developmental disabilities (such as tutors or resource teachers)?**				
Yes	120	65.9	47	63.5
No	22	12.1	14	18.9
I do not know	6	3.3	7	9.5
Did not respond	34	18.7	6	8.1
**During the past year, did your family have any difficulties or delays in getting educational services for your child for any of the following reasons?**				
There were no difficulties or delays	98	53.8	45	60.8
Delayed because of ineligibility	8	4.4	4	5.4
Delayed because of lack of information about services	7	3.8	5	6.8
Delayed because of lack of services in our area	5	2.7	3	4.1
Delayed because of long wait times	13	7.1	1	1.4
Delayed because of unreasonable costs	5	2.7	1	1.4
Delayed because the kindergarten/school did not want to enroll my child	22	12.1	5	6.8
Other	14	7.6	9	12.2
**Are there any local service centers that specialize in teaching parents the best ways to manage and support the needs of their children?**				
Yes	57	31.3	19	25.7
No	59	32.4	32	43.2
I do not know	44	24.2	17	23.0
Did not respond	22	12.1	6	8.1
**If so, do you currently use any of these services to learn more about managing and addressing the needs of your child?**				
Yes	43	23.6	14	18.9
No	47	25.8	22	29.7
I do not know about such services	44	24.2	19	25.7
Did not respond	48	26.4	19	25.7
**In the time since learning about your child’s disability, how difficult have you found it to obtain accurate and helpful information on the best ways to address your child’s needs?**				
Not difficult at all	21	11.5	4	5.4
Somewhat difficult	72	39.6	30	40.5
Very difficult	35	19.2	21	28.4
Extremely difficult	31	17.0	13	17.6
I have not tried to obtain information	1	0.5	−	−
Did not respond	22	12.1	6	8.1
**What do you consider to be the greatest challenges to caring for a child with developmental difficulties?** ***Please select the top three challenges from the list below.***				
Troubling behaviors (e.g., self-injury, aggression, tantrums)	66	36.3	29	39.2
Daily living skills (e.g., using the bathroom, dressing themselves, feeding themselves)	71	39.0	40	54.1
**Health problems (e.g., mental/physical health illnesses occurring alongside your child’s disability)**	14	7.7	21	28.4
Sleep problems (e.g., trouble falling asleep, trouble staying asleep)	35	19.2	17	23.0
**Diet/eating difficulties**	42	23.1	5	6.8
Social interaction difficulties (e.g., has difficulty making friends, can’t read social cues)	103	56.6	32	43.2
Repetitive behaviors/limited interests/insistence on sameness	65	35.7	15	20.3
Communication difficulties (e.g., cannot explain their needs, cannot express emotions)	89	48.9	23	31.1
Safety concerns (e.g., getting in trouble with police, neighbors, strangers)	44	24.2	11	14.9
Sensory issues (sensitivity to certain sounds or lights)	41	22.5	16	21.6
Other (Please specify: ______)	6	3.3	9	12.2
**What are the greatest challenges you face in getting support for your child?** ***Please select the top three challenges from the list below***				
Making sure my child receives adequate medical help	50	27.5	30	40.5
Making sure my child receives adequate education	115	63.2	45	60.8
Making sure my child receives adequate counseling help	86	47.3	25	33.8
Making sure my child’s basic rights are protected	73	40.1	25	33.8
Making sure my family and I receive adequate financial support	65	35.7	25	33.8
Making sure my family and I receive adequate support from the community	54	29.7	25	33.8
Other (Please specify: _________________)	7	3.8	4	5.4
**What do you consider to be the greatest priorities for families affected by developmental disabilities in your country?** ***Please select the top three priorities* from the list below.**				
Improved medical services	53	29.1	31	41.9
Improved education services	118	64.8	45	60.8
Improved counseling services	85	46.7	31	41.9
Greater rights for individuals with disabilities	63	34.6	27	36.5
Greater protection of existing rights for individuals with disabilities	56	30.8	16	21.6
**More information about autism/developmental delay**	75	41.2	16	21.6
Greater in-home support	24	13.2	12	16.2
Greater community awareness	81	44.5	33	44.6
Greater financial support for the family	77	42.3	23	31.1
Other (Please specify: ______)	−	−	3	4.1

The symbol * means that participants could choose more than one answer.

In terms of caregiver burden related to diagnosis, the majority of children (66.5%) received a diagnosis within their own town/village and additional 31.3% within the country. In terms of how long they had to wait from the initial pursuit of diagnosis to its ultimate confirmation, there was an even distribution across choices: 22% reported they waited less than a month, 29.1% reported between 1 and 3 months, 24.7% reported between 3 and 6 months, 9.3% between 6 months and a year, and 13.7% – over a year. The majority of caregivers (89.6%) had only one child with a developmental disorder in their household.

When reporting on their service experiences, 46.7% caregivers indicated that their family did not experience any difficulties or delays accessing medical services. However, 21.4% reported delays due to long wait times, 17% - due to lack of information about services, 10.4% - due to unreasonable costs, 17% – no services available in general or in their area, and 7.1% because of ineligibility. A similar pattern of responses was observed with regard to encounters with counseling services and educational services (see Table 4). There were no significant associations between caregivers’ education and their experienced delays and/or difficulties accessing medical, counseling, or educational services. There was a significant association between family income and access to educational services, however [χ^2^(2, *N* = 182) = 7.66, *p* = 0.022]. Caregivers with lower income were more likely to experience delays in educational resources.

When asked about the availability of local centers in their region providing trainings for parents, only 31.3% of respondents indicated that there were such. In addition, the majority of caregivers (76.4%) indicated that they had experienced difficulty in finding accurate and helpful information about addressing their child’s needs with 39.6% choosing that it was somewhat difficult, 19.2% that it was very difficult, and 17% that it was extremely difficult. There was no significant association between parent education and caregivers’ difficulty finding information.

We asked parents about their top three greatest challenges to caring for their child (see Table 4). The most common challenge picked by 56.6% of caregivers was social interaction difficulties, including difficulty making friends, and not being able to read social cues. This was followed by communication difficulties (48.9%), problems with daily living skills (39%), troubling behaviors (36.3%), and repetitive behaviors/limited interests (35.7%). Close to one-fifth of caregivers reported challenges with safety concerns (24.2%), their child’s sensory issues (22.5%), and sleep problems (19.2%).

When asked about caregivers’ greatest challenges in getting support for their child, the majority of respondents (63.2%) picked adequate education, followed by adequate counseling help (47.3%), and making sure that the child’s basic rights are protected (40.1%). Among the other popular choices were receiving financial support (35.7%) and adequate support from the community (29.7%). There were no significant associations between caregivers’ top three problems and top three challenges with their education or income.

In terms of greatest priorities for families affected by developmental disorders in Bulgaria, the majority of respondents in the ASD group (64.8%) similarly picked improved education services, followed by improved counseling services (46.7%), and greater community awareness (44.5%). These top three choices were followed by greater financial support for the family (42.3%), more information about the child’s condition (41.2%), and greater rights for individuals with disabilities (30.8%). There was no significant association between caregivers’ education and family income with the likelihood of choosing any of the priorities.

#### 3.3.2 oNDD group

See Table 4 for caregivers’ beliefs about the causes of their child’s condition, distanced traveled and wait time to receive an official diagnosis, and delays in getting medical, counseling, and educational services. There were no associations between caregivers’ education and their beliefs. There were no associations between caregiver education and family income and access to services or information.

See Table 4 for the top three challenges to caring for a child with a developmental disorder, the top three challenges to getting support for the child, and the top three greatest priorities for families. There were no associations between the top three challenges to care and the top three priorities with caregiver education and family income.

#### 3.3.3 ASD vs. oNDD comparison

Caregivers’ beliefs about the causes of their child’s condition differed across groups, [χ^2^(9, *N* = 256) = 37.780, *p* < 0.001]. Specifically, caregivers in the ASD group were more likely to select “vaccines” or “unknown causes” than caregivers in the oNDD group.

There were no statistically significant differences between diagnostic groups and distance traveled to receive a diagnosis. However, there was an association between diagnostic group and the wait time between initial pursuit of diagnosis and the confirmation of the diagnosis [χ^2^(5, *N* = 256) = 24.488, *p* < 0.001] with oNDD group receiving a diagnosis more quickly.

There were no significant differences between groups in service encounters, including access to and delay of medical, counseling, and educational services, and access to parent training and information.

Some differences emerged, however, in top three challenges to caring for a child with developmental difficulties. Specifically, the oNDD group was more likely to select “health problems” [χ^2^(1, *N* = 256) = 19.074, *p* < 0.001]. Furthermore, the ASD group was more likely to select “diet” as a problem [χ^2^(1, *N* = 256) = 9.349, *p* = 0.002].

There were no group differences in top three challenges to care, and there was only one group difference in top three priorities. The ASD group was more likely to select “more information about autism/developmental delay” than the oNDD group [χ^2^(1, *N* = 256) = 8.810, *p* = 0.003].

### 3.4 Open responses

The very last question on the survey was a comment box asking respondents to provide any comments, suggestions, and/or recommendations about what services should become available, but also about what the main priorities should be when changing policy related to families affected by developmental disorders. Out of the 317 respondents, 118 (37.2%) filled out the comment box. Responses ranged from a list of specialists that the caregiver wanted available for their child to paragraph-long discussion of the situation in Bulgaria and how the respondent envisioned existing policy should change.

**FIGURE 1:**
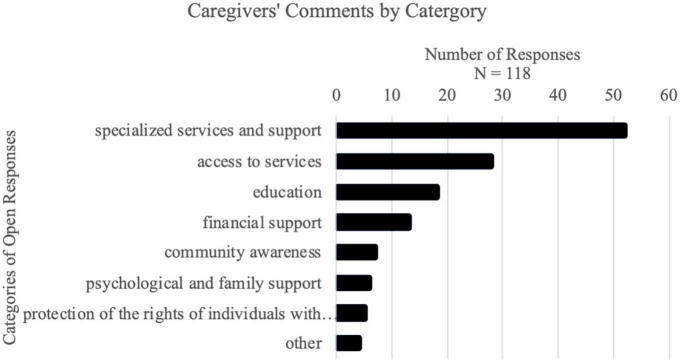
Distribution of caregivers’ comments by category.

We went through the responses and based on their content, categorized them into 8 distinct categories: “specialized services and support,” “access to services,” “education,” “financial support,” “community awareness,” “psychological and family support,” “protection of the rights of individuals with disabilities,” and “other.” The order of these categories reflects the number of responses classified under them, with “specialized services and support” containing the highest number of responses (see Figure 1). Each category is described in Table 5.

**TABLE 5:** Topics in open-ended comment box at the end of the survey.

Number of responses	Topic	Details
52	Specialized services and support	All responses that discussed the specific medical professionals and clinicians that the respondent considered lacking in the country. Common examples include SLPs, psychologists, occupational therapist, ABA therapist, etc. These responses often included exhaustive lists of medical professionals.
28	Access to services	All comments in which respondents wrote about how they envisioned they should receive services. Specifically, they described day centers that would provide all necessary services rather than having to take their child from professional to professional around the city. Another common theme was a consistent and individualized approach to each child across all service providers that they work with.
18	Education	All responses that talked about the need for better trained tutors and resource teachers, as well as the need to come up with a specialized curriculum for children with developmental disorders rather than following the curriculum of their typically developing peers.
13	Financial support	All responses that discussed the need for financial support for affected families, as well as the possibility of making services free or available at a reasonable cost. Some responses also mentioned the need to increase salaries of specialists providing services to children with developmental disorders.
7	Community awareness	All responses discussing the need for raising community awareness about developmental disorders. The responses described raising awareness both among peers and parents, but also among teachers and medical professionals.
6	Psychological and family support	All responses discussing the need for services and resources for parents/families, including support groups, resources for families right after they receive their child’s diagnosis, and struggling with loneliness.
5	Protection of the rights of individuals with disabilities	All responses that talked about the need for better ‘protection of the rights of individuals with disabilities.’ Responses mentioned the need for the “archaic system” to adapt to the needs of the children rather than the other way around, and for the need of the “country” to protect these children as they transition to adulthood.
4	Other	All responses that did not fall into any of the other categories.
		

Note that a comment could be classified under more than one category. The total number of comments was 118.

## 4 Discussion

This is one of the first studies collecting primary data from families of children with developmental disorders in Bulgaria aimed at characterizing their needs and daily burdens. This study has three main findings: (1) children with ASD and children with oNDD in Bulgaria have different needs and paths to diagnosis; (2) nevertheless, children in both groups experience similar challenges to accessing medical, counseling, and educational services, regardless of their demographic characteristics; and (3) parents’ priorities focus on education, counseling and medical support, protecting children’s basic rights, and raising awareness. Based on our findings, we provide specific recommendations for changes in services and policy.

### 4.1 Comparison of ASD and oNDD children’s needs and paths to diagnosis

Overall, the ASD group and the oNDD group did not differ in terms of demographic information. The majority of respondents were female between 36 and 45 years of age, primary caregivers and biological mothers of the child for whom they filled out the questionnaire. Our sample was highly educated with over 75% of respondents having received an undergraduate or a graduate degree compared to only 26.1% of Bulgarian population over the age of 25 years in 2020 that had attained at least a Bachelor’s degree (25). We attribute this characteristic of our sample to our recruitment strategy, namely relying on parent groups online where parents proactively seek out advice and try to discuss treatment and intervention practices. The higher education level of our respondents might introduce some bias to our results considering that the sample is not representative of the general population when it to comes to educational attainment. Nevertheless, there was more variability in terms of family income, where over one-third of respondents reported income lower than the country average monthly wage (24). Perhaps, this can be linked to our respondents’ employment status (46.6% were employed full-time or part-time), which was much lower than the 94% employment rate for the country in 2020 for people between the age of 20 and 64 years (24). The lower employment rate and lower income of our respondents but higher education than the national average could potentially be attributed to the fact that the majority of our respondents are the primary caregiver of their children with a developmental disorder, and the caregiving demands might be impacting their employment. Even though the two participant groups were comparable in demographic characteristics, they differed in terms of the characteristics of the children, their needs, and paths to diagnosis. Specifically, there were differences in caregivers’ reasons for first concern. The oNDD parents noticed motor difficulties first, while ASD parents were more likely to notice communication difficulties, social challenges, and restrictive and repetitive behaviors first. In addition, oNDD parents were more likely to express concern about the development of their child earlier, with 58% of them expressing first concern in the first year of the child’s life in comparison to only 16% of ASD parents. These differences in reasons for first concern and when it was expressed could be attributed to the different defining characteristics of the conditions across the two participant groups. Specifically, some oNDD and genetic syndromes have symptoms that might present immediately after birth. In contrast, differences in repetitive behaviors, cognition, and language between children with and without later ASD diagnosis have been found later by 14–16 months of age (26). Furthermore, physical/motor difficulties are perhaps easier to notice for parents without a training in early development than more subtle social and communication challenges that would start to affect the child’s behavioral repertoire later (as their communication abilities develop). In past studies, parents from the United States and the United Kingdom expressed first concern for their child by 14−15 to 19 months of age (27–29). In our ASD sample, first concerns were expressed somewhat later with 82.2% of ASD parents expressing first concern by 24 months. However, our results are comparable to what was reported for Bulgarian parents in the past (4) with a mean age of first concern of 24.7 months and for Serbian parents with a mean age of 22.5 months (30). Efforts to improve awareness about child development and expected developmental milestones among parents could help lower the age of first concern.

The later age of first concern, in turn, is expected to have cascading effects on the age of diagnosis. Indeed, oNDD children were more likely to receive a diagnosis earlier with 45.2% receiving it within first year of life as compared to only 1% of ASD children. oNDD children not only were diagnosed earlier, but they received a diagnosis faster with 79.5% of them waiting less than 3 months from first attempt in comparison to 50.3% of ASD children who waited less than 3 months. What could account for the different wait times to diagnosis across the ASD and oNDD groups? On the one hand, it could be the length of the diagnostic procedures that is different with some oNDD conditions requiring genetic testing and EEG testing, while an ASD diagnosis requires a more extensive battery of behavioral assessments over an extended period of time. Relatedly, the difference in wait times could be attributed to the number of specialists available and qualified to provide a diagnosis. In Bulgaria, only a child psychiatrist can provide a formal diagnosis of autism spectrum disorder based on the ICD-10. In contrast, conditions such as epilepsy, cerebral palsy, or genetic syndromes can be diagnosed by child neurologist and professionals specializing in genetic disorders. In comparison to past research, the average age of ASD diagnosis in North America is typically after 3 years of age and up to 5 and a half [(31) – 55.2 months; (32) – 38 months; (33) – 68 months; see (34) for review]. In Bulgaria over 5 years ago, the average age of ASD diagnosis was 46.6 months (4). These results are similar to the present study with 58.2% of ASD children receiving a diagnosis before 3 years and 37.9% between 4 and 8 years of age.

Another comparison to previously collected data in Bulgaria shows a relative improvement in diagnostic services. Based on data collected between 2013 and 2017, 50% of Bulgarian parents with ASD had to travel more than 100 km to receive a diagnosis and 17% had to travel between 25 and 100 km (4). In contrast, our results show that over 60% of parents across both the ASD and oNDD groups received a diagnosis within their own town or city. This improvement could be attributed to more diagnostic services becoming available or due to differences in the sampling of the two studies.

In summary, the diagnostic experiences of children with ASD and oNDD are different and so should be addressed accordingly. In particular, work needs to be done in raising awareness about developmental disorders, and in providing earlier and faster identification and diagnosis, especially for children with ASD.

### 4.2 Access to medical, counseling, and educational services

Regardless of the group differences in diagnostic experiences, both groups experienced similar delays in accessing services. Close to half of all children across both groups experienced delays in accessing medical, counseling, and educational services. These results support the evaluation of available services by the National Network for Children in their yearly report card (15). The reported reasons for the delays in access were somewhat evenly distributed across issues with eligibility, lack of information about such services, lack of existing services in the area, long wait times, and unreasonable costs. A notable exception was the long wait times associated with getting medical services, where 20.5% of children in the ASD group and 17.8% of children in the oNDD group experienced long wait times. Therefore, even though these children get diagnosed at different ages and for a different duration, when it comes to accessing services, they are similarly disadvantaged. The challenges associated with accessing services could be addressed by making more services available, thus reducing wait times, subsidizing costs, and generally promoting information about services and who is eligible for them. In addition, other efforts could include providing physicians with more information and training about developmental differences.

In addition to challenges associated with accessing services for their children, there were similar challenges across groups associated with access to parent trainings. In both the ASD and oNDD groups, less than one-third of parents (31.8% in the ASD group; 26% in the oNDD group) reported that there were any local centers teaching parents about the best ways to support the needs of their children. This issue of the lack of availability of parent trainings could have two related interpretations. On the one hand, the medical model of disability, which dominated the diagnostic process and educational services for children with developmental disorders and disabilities in the past (8), could account for the lack of services that are targeting the parents as the means for providing support for their children. This, in turn, could also lead to fewer parents even looking for such resources based on the expectation that it is only specialists who can support the development and functioning of their children. On the other hand, it could be the lack of parent training models available that are translated and adapted to the Bulgarian context that accounts for this finding. In fact, just recently the ImPACT training (35) was formally introduced in the country as part of the Stay-In Project co-funded by the Erasmus + Programme of the European Union (36). Data on parents’ inclusion in already existing early intervention services showed that less than 10% of parents were present in the room with their child and actively trying out different treatment strategies (16), once again echoing the need for approaches engaging and training the parents. Engaging parents in applying treatment and intervention practices at home is perhaps the most scalable approach to reach the highest number of children, especially in contexts where there are not enough trained professionals and counseling services available.

Parents not only did not have access to parent training services but also reported difficulty finding helpful information on how best to address their children’s needs. Specifically, 76.4% of ASD parents and 86.3% of oNDD parents found it somewhat difficult, very difficult, or extremely difficult to obtain useful information. Perhaps, this could partially be attributed to the fact that upon receiving a diagnosis, parents are not formally provided with guidance and materials about their child’s condition and about what, where, and how to seek services. Parents, as the ones who spend most time with their children during childhood, have the potential to implement treatment and intervention strategies with their children on a daily basis. Supporting parents by providing them with freely available and accessible information about how to best promote their children’s development is one very easy and cost-effective way to start addressing their needs and the general lack of services in low-resource contexts. In addition, raising awareness about developmental disorders and improving access to information could help improve caregivers’ understanding of the etiology of developmental disorders, which should be a key goal considering that in our sample over 30% of parents selected vaccinations as the cause of their child’s ASD diagnosis. When planning awareness campaigns, it is essential to not only present evidence-based advice but also to point out what the criteria are for a publication to be considered reliable and valid.

### 4.3 Parents’ challenges and priorities

One way to move away from the strictly medical approach to developmental disorders and disabilities is to start addressing the social, communicative, and daily living needs of the children, as well as to treat them and their families as a unit. The third main finding of this study pertains specifically to the challenges, needs, and priorities of the families and could be used to build data-driven strategies to improve local services.

In our sample, parents were typically the first to notice and express concern about their child’s development and it was their desire to help their child that motivated them to seek a formal diagnosis. Considering that parents are the ones who spend the most time with their children in early childhood, it would be beneficial to raise awareness among parents about typical developmental milestones in their children’s functioning to lower the age at which parents express a first concern about their child’s development. However, once the child has received a formal diagnosis, what are parents’ greatest challenges to care? For the majority of parents in our sample (across both groups), the top three challenges to care were social interaction difficulties, communication difficulties, and daily living skills. Similar results were found from parents of children with ASD from Serbia (30) and from Low and Low Middle Income Countries (LMIC) in South America (37). Although social interaction and communication difficulties are often addressed in already available SLP and counseling services, parents in their open responses discussed the need for other services such as occupational therapy and applied behavioral analysis (ABA) therapy that could potentially improve the daily living skills of their children. Therefore, programs need to be developed that focus specifically on improving the daily functioning of children as it pertains to getting dressed, feeding, brushing their teeth, and doing chores. It should be pointed out that oNDD parents indicated that their children’s health problems were a key challenge, while ASD parents selected their children’s diet/eating difficulties. Furthermore, more oNDD parents experienced challenges in getting medical help for their children, which could be attributed to the nature of their diagnosis and associated medical conditions. In contrast, more ASD parents experienced challenges in receiving counseling help, again alluding to the different symptoms of the children, which translate into different needs. These differences in challenges should be accounted for in the way specific services and policies are made for children with ASD and children with oNDD.

In terms of parents’ greatest priorities, over 60% of caregivers across both groups selected receiving adequate education. This key priority has been reported by parents in LMIC in Southeast Europe (4, 30) and in South America (37), as well as by parents in high-income countries (38). Education is one of the primary factors associated with positive long-term outcomes in children with ASD (39). Furthermore, education is perceived as children’s path to socializing with their peers and to becoming independent. Parents valuing their children’s education was further reflected in their open-ended responses, in which they discussed the need for better trained tutors and resource teachers, as well as for developing a specialized curriculum for children with developmental disorders. Therefore, even though progress has been made in education in Bulgaria over the last year (15), there is still more to be done to adequately support students with developmental disorders in the country.

In addition to education as a main challenge and a priority, caregivers also selected the protection of their children’s basic rights and raising community awareness. Although community awareness has not been identified as a key priority for parents in high-income countries (39, 40), it has been identified as a key priority by over 40% of Bulgarian parents in parents our sample, as well as by parents from low- and middle-income countries in South America (37). Therefore, in addition to improving specific services for children with developmental disorders, there is a need to improve community awareness about developmental disorders. In their open-ended responses, caregivers discussed the need for raising awareness not only among other parents but also among teachers and medical professionals. A similar need for awareness has been found in relation to education suggesting that the majority of parents are not familiar with the needs of children with developmental disorders and thus are less likely to support their inclusion in the classroom (9). Raising community awareness will not only affect attitudes toward inclusive education but could also reduce stigma for families, which is frequently experienced by families of children with ASD (41, 42).

In addition to education, protection of children’s basic rights, and raising community awareness, parents expressed other priorities. Specifically, caregivers envisioned that their children should receive services following an individualized and unified approach across professionals (SLP, counseling, OT, etc.). Furthermore, over one-third of all respondents identified the need for greater financial support, which is a reflection of the financial burden associated with raising a child with a developmental disorder. Therefore, future policy should also address the coordination between institutions and professionals working with the child, as well as the financial aspects associated with accessing services.

### 4.4 Limitations

Although very informative, the present study possesses a number of limitations. First, because there is no population-based sampling of children with developmental disorders in Bulgaria, we relied on a convenience sample. We recruited respondents on social media, through online parent forums and websites, and through listservs from centers providing services for children with developmental disorders. Furthermore, in the analyses, we only included families, whose child had a confirmed diagnosis of a developmental disorder. Therefore, our sample (1) might not be representative of the general population in the country, (2) could be subject to selection bias with more parents already seeking information and services for their children being more likely to participate, and (3) might not be representative of children who have not received a diagnosis yet or whose families are unwilling to seek one. Another limitation of our sample is that the caregivers of children with autism spectrum disorders were over-represented as they made up 73% of all respondents. Nevertheless, caregivers in the oNDD group had children with a wide range of neurodevelopmental disorders reflecting the heterogeneity of these conditions. Yet, another limitation is that the majority of respondents were the children’s biological mothers and thus they might offer a different perspective on the needs of their children than other family members. Future studies should focus on sampling more caregivers of children with oNDD disorders to be able to investigate their specific needs based on diagnosis. Another limitation to our study is that children’s diagnoses were not verified by a clinician, so we are relying on the accuracy of parent report. Furthermore, all responses were based on self-report and so could be subject to interpretation and be affected by the fact that parents were reflecting on past events.

Another limitation that needs to be addressed in the future is about children’s characteristics, specifically age, sex, native language, etc. As of now, there are still no publicly available data on the number of children with developmental disorders in Bulgaria and their breakdown by diagnosis. Such information will help inform policy about the number of professionals and kinds of services that need to be made available to address the needs of these children and their families.

## Data availability statement

The datasets presented in this article are not readily available because according to our Committee for Research Ethics application, we have not obtained permission to do that. Requests to access the datasets should be directed to MB, mihaela.barokova@gmail.com.

## Ethics statement

The studies involving human participants were reviewed and approved by the Committee for Research Ethics of Department of Cognitive Science and Psychology at New Bulgarian University. The patients/participants provided their written informed consent to participate in this study.

## Author contributions

MB and AA-S organized the data collection. MB and AK worked on data processing and analysis. MB drafted the first version of the manuscript. All authors participated in the conception and design of the study and contributed to the final written version of the manuscript.

## Footnotes

1 In the paper, the label “developmental disorder” is used to represent all formal diagnoses of a developmental delay, disorder, and/or disability as defined by the International Classification of Diseases.
